# Selective Loss of TGFβ Smad-Dependent Signalling Prevents Cell Cycle Arrest and Promotes Invasion in Oesophageal Adenocarcinoma Cell Lines

**DOI:** 10.1371/journal.pone.0000177

**Published:** 2007-01-31

**Authors:** Benjamin A. Onwuegbusi, Jonathan R.E. Rees, Pierre Lao-Sirieix, Rebecca C. Fitzgerald

**Affiliations:** MRC Cancer Cell Unit, Hutchison-MRC Research Centre, Cambridge, United Kingdom; Dresden University of Technology, Germany

## Abstract

In cancer, Transforming Growth Factor β (TGFβ) increases proliferation and promotes invasion via selective loss of signalling pathways. Oesophageal adenocarcinoma arises from Barrett's oesophagus, progresses rapidly and is usually fatal. The contribution of perturbed TGFβ signalling in the promotion of metastasis in this disease has not been elucidated. We therefore investigated the role of TGFβ in Barrett's associated oesophageal adenocarcinoma using a panel of cell lines (OE33, TE7, SEG, BIC, FLO). 4/5 adenocarcinoma cell lines failed to cell cycle arrest, down-regulate c-Myc or induce p21 in response to TGFβ, and modulation of a Smad3/4 specific promoter was inhibited. These hyperproliferative adenocarcinoma cell lines displayed a TGFβ induced increase in the expression of the extracellular matrix degrading proteinases, urokinase-type plasminogen activator (uPA) and plasminogen activator inhibitor 1 (PAI-1), which correlated with an invasive cell phenotype as measured by *in vitro* migration, invasion and cell scattering assays. Inhibiting ERK and JNK pathways significantly reduced PAI and uPA induction and inhibited the invasive cell phenotype. These results suggest that TGFβ Smad-dependent signalling is perturbed in Barrett's carcinogenesis, resulting in failure of growth-arrest. However, TGFβ can promote PAI and uPA expression and invasion through MAPK pathways. These data would support a dual role for TGFβ in oesophageal adenocarcinoma.

## Introduction

Transforming Growth Factor β (TGFβ), and the components of its signal transduction pathway, are known to demonstrate tumour suppressor activity. In many human cancers, there are inactivating mutations in components of the TGFβ pathway resulting in uncontrolled proliferation. In addition, through its actions on both the tumour cells and the surrounding stromal cells, TGFβ can enhance invasion, motility and metastasis. TGFβ can thus play a dual role in the initiation and malignant progression of human cancer [1], [2].

TGFβ signalling occurs through activation of type I and II trans-membrane serine/threonine kinase receptors, which leads to the phosphorylation of Smad 2 and 3. This complex, in association with Smad 4 translocates to the nucleus resulting in transcriptional activation of downstream targets [3]. The anti-proliferative effects of TGFβ in normal epithelial cells are achieved through the inactivation of cyclin dependent kinases (cdk) 2, 4 and 6, which lead to cell cycle arrest. This is mediated by the rapid induction of the cdk4/6 inhibitor p15^INK4B^, and the cdk2 inhibitor p21^CIP1/WAF1^. Another key event in TGFβ mediated cell cycle arrest involves the repression of c-Myc. This transcription factor binds the promoters of p15^INK4B^ and p21^CIP1/WAF1^ and suppresses their expression [4]. Rapid transcriptional down-regulation of c-Myc by TGFβ relieves this repression, allowing binding of the heteromeric Smad complex [5]. The net effect of this is to halt the cell cycle in G_1_, and thus potently inhibit proliferation [6].

TGFβ is frequently overexpressed by epithelial cancer cells which become unresponsive to its anti-proliferative effects and this leads to paracrine stimulation of stromal cells within the tumour microenvironment [6]. As a result there is stimulation of angiogenesis and upregulation of extracellular matrix (ECM) degrading proteinases. Expression of the urokinase-type plasminogen activator (uPA) is upregulated by TGFβ [7], which through the formation of plasmin enables the tumour cell to penetrate the basement membrane. Overexpression of uPA has been observed in invasive malignancies of the breast, colon and stomach [8]. The uPA inhibitor plasminogen activator inhibitor 1 (PAI-1) is another TGFβ target gene that is up-regulated in advanced cancers [8]. The paradox of an elevated expression level of PAI promoting tumour invasion is partially explained by the observation that this inhibitor can promote cell migration and angiogenesis, independent of the effects on plasmin activity [9], [10].

In contrast to the plethora of information about mutations in the TGFβ signal cascade in colon, gastric and pancreatic cancers [2], [11]–[13], the contribution of perturbed TGFβ signalling in oesophageal adenocarcinoma has not been elucidated completely. Oesophageal adenocarcinoma usually arises through a multi-step sequence from Barrett's metaplasia to dysplasia and carcinoma [14]. Recent *in vivo* and *in vitro* evidence suggests that inactivation of SMAD4, lack of RUNX3 and inability to degrade SnoN may be some of the causes for the unresponsiveness of oesophageal cancer to TGFβ anti-proliferative effect [15]–[17]. This therefore begs the question whether TGFβ can promote invasion in this disease in the context of deranged SMAD4 signalling.

The signalling pathways by which TGFβ exerts its effects on migration and invasion are gradually being elucidated. The activated TGFβ receptor complex is now known to activate kinase pathways independently of Smad signalling and these have been shown to stimulate the expression of both uPA and PAI [18]–[20]. These pathways include the phosphoinositol-3 kinase (PI3K)[9], and the mitogen activated protein kinases (MAPK) particularly extracellular signal regulated kinase (ERK),[21] *c-jun* N-terminal kinase (JNK) [22], and p38 [23].

The specific aims of this study were therefore: 1). To determine whether TGFβ can simultaneously affect proliferation (cell cycle, c-Myc and p21 expression) and invasion indices (ECM proteinase expression and functional invasive characteristics) in oesophageal adenocarcinoma cell lines; 2). To elucidate the cell signalling pathways involved in these responses.

## Results

### Lack of anti-proliferative response to TGFβ

**Figure 1:**
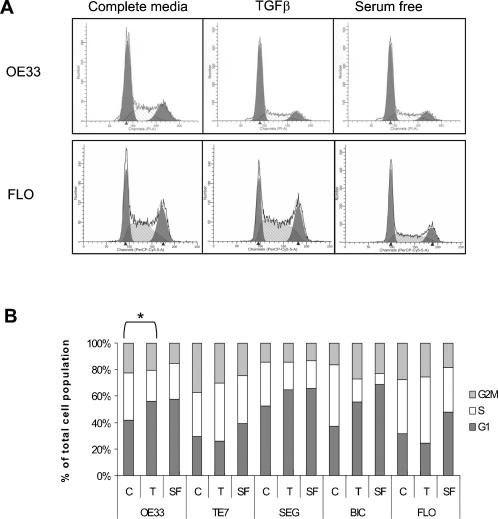
Effect of TGFβ on cell cycle progression. All cells were synchronised overnight in serum free media. Cells were then released into cell cycle by complete media (C), or kept continuously in serum free media (SF), or in complete media with TGFβ (10 ng/mL), all for 24 hours. DNA content was then assessed by flow cytometry. (A) Representative FACScan profiles for OE33 and FLO. The initial peak represents the G0/G1 fraction, whilst the second peak represents G2M fraction. (B) Summary of cell cycle distribution for each cell line analysed. Each bar represents mean percentage of total cell population in G0/G1 (grey), S (black) and G2/M (white) phase of the cell cycle from three separate experiments. * p<0.05, *** p<0.001

**Figure 2:**
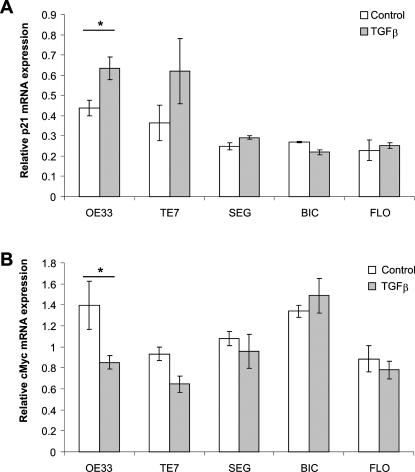
Regulation of anti-proliferative genes by TGFβ in oesophageal cell lines. mRNA expression of p21 (A) and c-Myc (B) was assessed by quantitative real-time PCR in control cells grown in complete media or in complete media containing 10 ng/mL TGFβ for 24 hours. Results for real-time PCR are expressed as the mean and standard error of four separate experiments relative to β-actin expression. * p<0.05, ***p<0.001

First, the effect of TGFβ on cell cycle progression and expression of cell cycle associated genes, p21 and c-myc, was assessed. The control experimental conditions were cells treated with serum free media (SF), which is known to induce cell cycle arrest, and cells treated with SF media followed by release into the cell cycle by complete media (C), (Figure 1A and 1B). All cells maintained in serum free media for 24 h demonstrated an increase in the proportion of cells in G0/G1 (Figure 1A, 1B). The cells were also arrested by stimulation with UV light as determined by a BrdU incorporation assay (data not shown). However, when cultured in the presence of TGFβ (T) only 1/5 BE adenocarcinoma cell lines (OE33), exhibited an increase in the fraction of cells in G0/G1 (11% OE33 p<0.05), (Fig 1A, 1B). Since the inhibition of proliferation in response to TGFβ would be expected to occur as a result of an up-regulation of p21^CIP/WAF1 ^and a decreased expression of c-Myc, the effect of TGFβ on these mRNA expression levels was examined. Real time PCR demonstrated that only OE33 cells exhibited a significant increase in p21 and decrease in c-Myc expression (Figure 2A and 2B), (p<0.05 in both cases). TGFβ is thus unable to significantly alter the expression of c-myc and p21 mRNA, and halt cell cycle progression in 4/5 BE invasive adenocarcinoma cell lines. This is in keeping with our previous data on cell proliferation measured using the MTT assay [17].

### Impaired Smad-dependent transcriptional regulation

**Figure 3:**
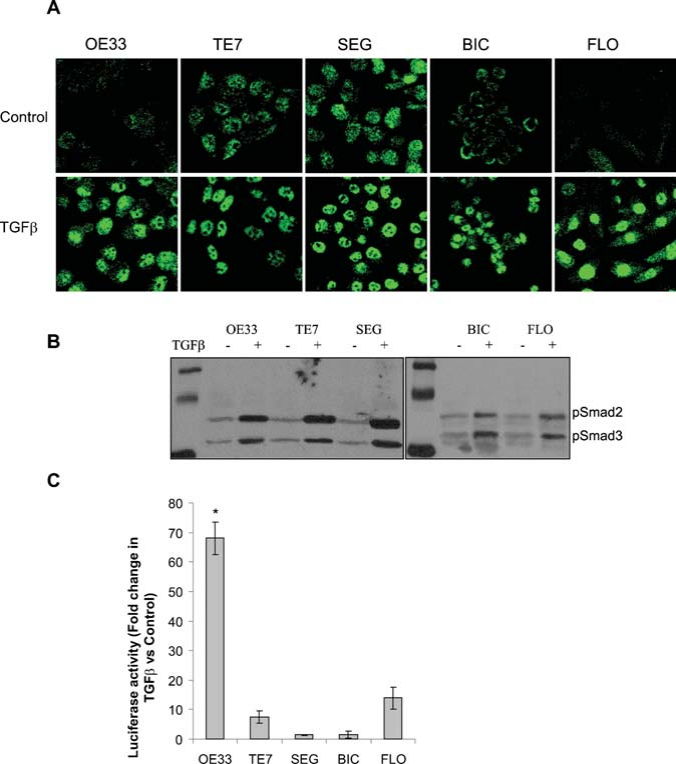
TGFβ stimulation of Smad pathway in oesophageal cell lines. Nuclear translocation (A) and phosphorylation (B) of Smad 2/3 following TGFβ stimulation. Cells were treated with 10 ng/mL of TGFβ1 for 6 h and Smad 2/3 localisation and phosphorylation were determined by immunofluorescence using anti-Smad 2/3 antibody and confocal microscopy and western blotting respectively. Regulation of transcription by TGFβ (C). Cells were co-transfected with the (CAGA)_12_-Luciferase reporter plasmid and the Renilla Luciferase reporter plasmid then incubated, with or without 10 ng/mL TGFβ for 24 h. Data is expressed as mean fold change in CAGA luciferase activity in TGFβ samples compared to untreated samples, normalised to the activity of Renilla, from four separate experiments * p<0.05, ** p<0.01, ***p<0.001

Nuclear translocation of Smad 2/3 (Fig 3A) and phosphorylation of Smad 2 and 3 (Fig 3B) were observed in all cell lines following stimulation with TGFβ. Nuclear accumulation of Smad2/3 in BIC, which does not express Smad4, confirms early work suggesting that Smad4 is not necessary for Smad 2/3 nuclear translocation [3]. The nuclear translocation of Smad 2/3 and of the phosphorylation of Smad 2 and 3 attest to the functionality of the TGFβ pathway. However, the cells remain unable to decrease their proliferation as a result of TGFβ stimulation. Therefore, to determine whether the inability of TGFβ to regulate the cell cycle is secondary to a lack of Smad dependent transcriptional regulation we used a (CAGA)_12_-luciferase reporter. The lack of Smad 4 expression in BIC makes this cell line the perfect negative control for transcriptional regulation of the TGFβ pathway. BIC and SEG both display no transcriptional activity while the low transcriptional activity in TE7 and FLO may not be sufficient to trigger an anti-proliferative response. Only the TGFβ responsive cells, OE33, demonstrated a marked induction of transcriptional activity in TGFβ treated cells compared to control cells (Figure 3C) (68±5.5; p<0.01). Hence, the non-responsiveness to the anti-proliferative effects of TGFβ may be due, at least partly, to a lack of Smad-dependent transcriptional regulation, which might stem from Smad4 inactivation, the inability to degrade SnoN and possibly other unknown mechanisms [16], [17].

### MAPK dependent TGFβ induction of genes associated with ECM regulation

**Figure 4:**
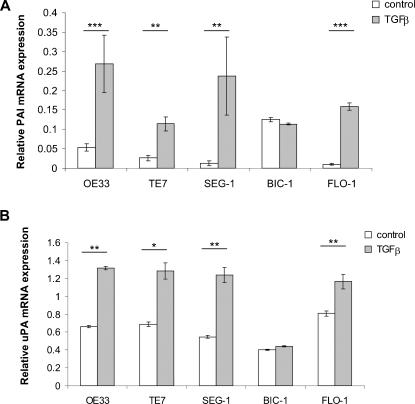
Regulation of ECM modulating genes by TGFβ. mRNA expression of PAI-1 (A) and uPA (B) was assessed by quantitative real-time PCR in control cells and cells treated with 10 mg/mL TGFβ for 24 hours. Results for real-time PCR expressed as mean and standard error of four separate experiments relative to β-actin expression. * p<0.05, ** p<0.01, ***p<0.001

The ability of TGFβ to regulate the genes uPA and PAI, which are associated with ECM modulation, was then assessed. All cell lines, with the exception of BIC up-regulated either PAI and/or uPA in response to TGFβ. (Figure 4A and 4B; OE33, FLO p<0.001; TE7, SEG p<0.05 for PAI and OE33, SEG, FLO p<0.01; TE7, p<0.05 for uPA). TGFβ was thus able to up-regulate the expression of both genes associated with ECM modulation in 4/5 cell lines, including three lines (TE7, SEG and FLO) that lacked Smad-dependent transcriptional responses and growth suppression by TGFβ.

**Figure 5:**
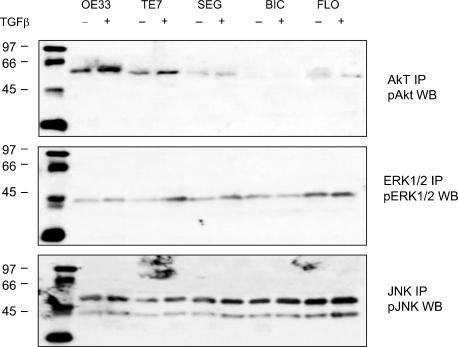
TGFβ modulation of kinase pathways in BE adenocarcinoma cells. Western blot on lysate from OE33, TE7, SEG, BIC and FLO cells treated with control media (−) or media with 10ng/mL TGFβ (+) for 4 hours for phosphorylated ERK1/2, phosphorylated JNK or phosphorylated Akt. Immunoprecipitation was performed with total antibody for Akt, ERK and JNK, and then blots were probed with the phosphorylated antibody.

To characterise the molecular basis for the differential responses of the cell lines to TGFβ, we analysed some of the Smad independent kinase pathways known to activate genes associated with ECM.[22] Overall, the levels of phosphorylated JNK did not vary following TGFβ treatment (Figure 5). The levels of phosphorylated Akt and ERK1/2 were increased in TE7 (1.6 and 5.3 fold respectively) and SEG (1.7 and 1.8 fold respectively) following 4 hours of stimulation with TGFβ (Figure 5 and Figure S1 shows densitometry data). Only pAKT was increased in OE33 (1.8 fold) following treatment but very little variation was observed in BIC and FLO (figure S1). There was no change in total expression levels of these kinases (data not shown). BIC cells did not show an induction of uPA, PAI and Smad independent kinase pathways and were therefore not studied further.

**Figure 6:**
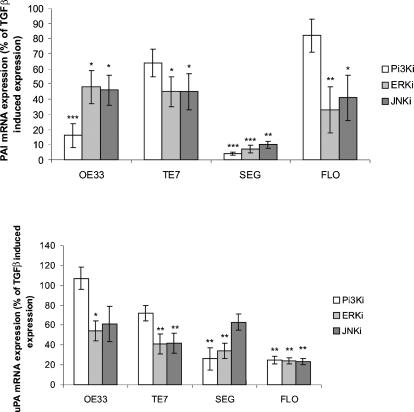
Effect of inhibition of PI3K, ERK and JNK pathways on PAI and uPA expression. Quantitative real-time PCR showing PAI (A) and uPA (B) expression in cells treated with 10 ng/mL TGFβ and PI3K inhibitor LY29004 (LY, 20 µM); ERK inhibitor PD98059 (PD, 10 µM); and JNK inhibitor SP600125 (SP, 10 µM) for 24 hours. Results are expressed as a percentage of expression in inhibitor treated samples compared to TGFβ treated samples, such that the greater the inhibition the lower the percentage expression. Results are mean and standard error of four separate experiments. * p<0.05, ** p<0.01, ***p<0.001

Inhibition of PI3Kactivity by LY294002 resulted in a variable effect on PAI expression (Fig. 6A). Specifically, in OE33 and SEG, TGFβ induced up-regulation of PAI mRNA was significantly diminished by LY294002 (OE33, SEG p<0.001). Inhibition of ERK activity by PD98059 significantly diminished TGFβ induced up-regulation of PAI mRNA in all cell lines (OE33, TE7 p<0.05; SEG p<0.001; FLO p<0.01), (Figure 6A). Following inhibition of JNK activity by SP600125 there was a reduction of the TGFβ induced up-regulation of PAI by a minimum of 50% in all cell lines analysed (p<0.05 for all cell lines). These findings were generally confirmed for a second of each class of kinase inhibitor (wortmannin, U0126 and Curcumin), (table S1), although ERK inhibition with UO126 was less effective than PD98059 possibly because of differential specificity of these inhibitors for MEK1 and 2. A similar pattern was seen when uPA expression was assessed in inhibitor treated cells (Figure 6B). Whereas, the effects of PI3K inhibition had a variable effect on uPA expression, inhibition of ERK by PD98059, and inhibition of JNK by SP600125 markedly reduced (by a minimum of 40%) the TGFβ induction of uPA in all BE adenocarcinoma cells (Figure 6B). Overall, the most consistent results were seen with the JNK inhibitors since both inhibitors (SP600125 and Curcumin) significantly reduced the up-regulation of PAI and uPA in all cell lines, (table S1).

**Figure 7:**
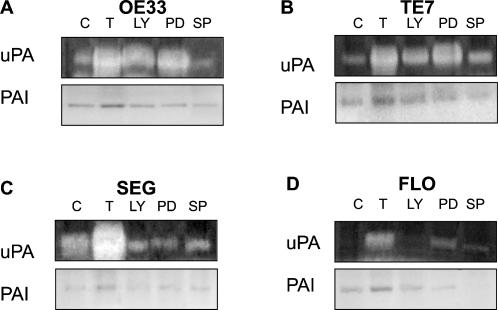
Effect of inhibition of PI3K, ERK and JNK pathways on PAI and uPA activity. uPA enzyme activity was assessed by casein zymography, and PAI activity determined by reverse casein zymography in cell lysates from untreated control cells (C), cells treated with 10 ng/mL TGFβ alone (T) and in cells treated with TGFβ and PI3K inhibitor LY294002 (LY), ERK inhibitor PD98059 (PD) or JNK inhibitor SP600125 (SP) for 24 h. uPA activity was detected as digested clear bands on a dark background, whilst PAI activity was detected as dark undigested bands against a clear background.

Next we determined whether inhibition of these kinase pathways had an effect on PAI and uPA enzyme activity, as well as expression levels. Zymography and reverse zymography indicated that TGFβ induces an increase in the proteolytic activity of uPA (increase in the white band intensity), and an increase in the ability of PAI to retard the action of uPA in all cells lines analysed (increase in the dark band intensity), (Fig 7A–D, compare lane C with T). The inhibition of PI3K activity by LY294002, and inhibition of ERK signalling by PD98059 resulted in a variable effect on uPA and PAI activity depending on the cell line (densitometry given as figure S2). Inhibition of JNK by SP600125 resulted in the most consistent decrease in activity of uPA and PAI (Figure 7A–D, compare lane T with SP for each cell line with a variation between 0.1 and 0.5 fold on densitometric analysis, figure S2). The expression and the enzyme activity data thus suggests that activation of the ERK and JNK pathways is important for TGFβ induced up-regulation of PAI and uPA in BE adenocarcinoma cells.

### TGFβ increases invasive cellular properties via activation of kinase pathways

We determined the ability of TGFβ to influence the invasiveness of cells using three *in vitro* assays: aggregation, invasion and wounding. An aggregation assay measures the ability of cells to grow in an anchorage-independent manner; invasion assays through matrigel measures the ability of cells to degrade the basement membrane and to migrate through it while the wounding assay only measures the ability of cells to migrate to occupy an empty space. Therefore a highly invasive cell *in vitro* should not form aggregates, should migrate through matrigel and to close up a wound. TGFβ had significant effects on at least one assay for each of the adenocarcinoma cells lines, compared to the control (no TGFβ), (Table 1). In order to examine the effect of kinase inhibitors these were only used in the cells lines exhibiting an increase in their invasive phenotype for a particular assay in response to TGFβ.

**Table 1:**
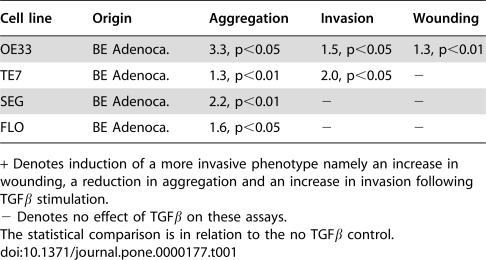
Summary of the invasive characteristics of the cell lines

Cell line	Origin	Aggregation	Invasion	Wounding
OE33	BE Adenoca.	3.3, p<0.05	1.5, p<0.05	1.3, p<0.01
TE7	BE Adenoca.	1.3, p<0.01	2.0, p<0.05	−
SEG	BE Adenoca.	2.2, p<0.01	−	−
FLO	BE Adenoca.	1.6, p<0.05	−	−

+ Denotes induction of a more invasive phenotype namely an increase in wounding, a reduction in aggregation and an increase in invasion following TGFβ stimulation.

− Denotes no effect of TGFβ on these assays.

The statistical comparison is in relation to the no TGFβ control.

**Figure 8:**
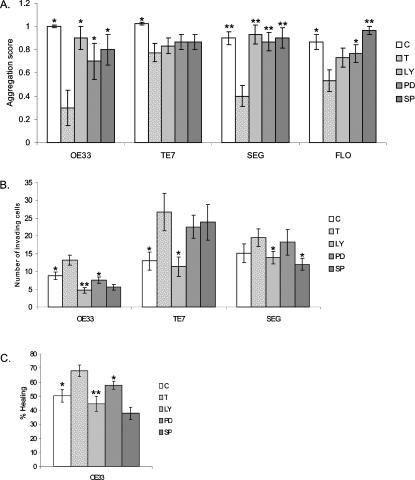
Effect of inhibition of PI3K, ERK and JNK pathways on invasive properties of oesophageal adenocarcinoma cells following TGFβ stimulation. (A) Aggregation in control cells cultured with normal medium (C), cells treated with TGFβ alone (T) or in the presence of PI3K inhibitor LY294002 (LY), ERK inhibitor PD98590 (PD) or JNK inhibitor SP600125 (SP). Scores represent the mean for 3 separate experiments where 0 is for no aggregates, 1 for small aggregates and 2 for large aggregates. (B) Invasion assay through matrigel matrix over 24 h in untreated cells (C), treated with TGFβ alone (T) or in the presence of inhibitors (LY, PD or SP), (C) Wound healing measured as the percentage of healing of a circular wound over 24 h was assessed in OE33 cells cultured in normal medium (C), or with the addition of TGFβ (T) or with TGFβ in the presence of inhibitors (LY, PD or SP). For all experiments TGFβ is compared with the control and the effect of inhibitors compared with TGFβ. * p<0.05, **p<0.01.

Therefore, aggregation was performed for TE7, SEG, FLO and OE33, invasion for TE7, SEG and OE33 and wounding for OE33. With regards to aggregation, the presence of all three kinase inhibitors diminished the cell scattering ability (a higher score denotes less scattering) of TGFβ in all the cell lines to a greater or lesser extent (Figure 8A). The increase in TGFβ-induced invasion observed in TE7, SEG and OE33 cells was significantly inhibited by LY294002 in TE7 cells (p<0.05), by SP600125 in SEG (p<0.05) and by all three inhibitors in OE33 (Figure 8B; p<0.001, LY294002; p<0.01, PD98059; p<0.001, SP600125). In OE33, the TGFβ induced increase in wound healing over 24 h (p<0.05), is significantly diminished by all three kinase inhibitors (Figure 8C; p<0.05). Overall inhibition of the JNK pathway with SP600125 had the most consistent effect seen in SEG, FLO and OE33 in all three of the assays.

## Discussion

These data suggest a dual role of TGFβ in the regulation of both the proliferative and invasive properties of BE adenocarcinoma *in vitro*. 4/5 BE cell lines are unable to mount an anti-proliferative response to TGFβ. However, the loss of TGFβ responsiveness is selective, such that the same cell lines that fail to exhibit an anti-proliferative response demonstrate a TGFβ dependent increase in the expression of PAI and uPA via PI3K, ERK and JNK (Table 2). The effect of kinase inhibitors on expression levels and the enzyme activity of uPA and PAI suggest that the ERK and JNK MAPK pathways may play a role. TGFβ induced activation of kinase pathways is functionally significant since their inhibition results in a reduction of cellular invasive properties *in vitro*.

**Table 2:**
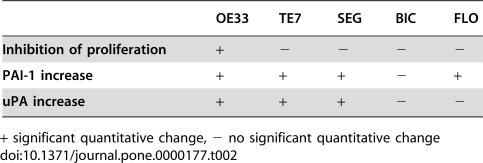
Modulation of proliferation and ECM associated genes by TGFβ in oesophageal cells

	OE33	TE7	SEG	BIC	FLO
**Inhibition of proliferation**	+	−	−	−	−
**PAI-1 increase**	+	+	+	−	+
**uPA increase**	+	+	+	−	−

+ significant quantitative change, − no significant quantitative change

TGFβ unresponsiveness in the BE adenocarcinoma cells could be attributed to an inability to down-regulate c-Myc, similar to breast cancer cells [24]. Given that all of the unresponsive cells in our panel do not demonstrate a TGFβ induced increase in transcriptional activity of a TGFβ reporter plasmid, defective Smad-mediated signal transduction is a possible underlying mechanism (Figure 3). However, since the Smad3/4 reporter construct is not the native promoter and may miss possible enhancer or suppressor elements this conclusion must be interpreted with caution. Furthermore, other mechanisms such as the inability to degrade SnoN have been described in oesophageal cells as a possible reason for a lack of responsiveness to TGFβ [16].

In contrast, there was a modest TGFβ dependent activation of kinase pathways (PI3K, ERK and JNK MAPK) in BE adenocarcinoma cells (Figure 5). This is interesting since inhibition of PI3K, ERK and JNK MAPK decreased the TGFβ induced expression of the ECM genes PAI and uPA (Figure 6). The PAI promoter contains motifs complementary to the binding sequence of AP-1 [25], [26], while the uPA promoter also contains two AP-1 binding sites [27]. Targets of the MAPK pathways ERK and JNK include the transcription factors c-Fos, c-Jun and ATF-2, which can undergo c-Fos/c-Jun or c-Jun/ATF-2 heterodimerisation to form AP-1. Activation of MAPK pathways can thus increase the expression of PAI and uPA [28], [29]; however, we cannot rule out the contribution of other pathways such as matrix metalloproteinases which also contain AP-1 promoter sites and are involved in ECM remodelling during invasion.

The combination of uPA, uPA receptor and PAI is crucial in the modulation of ECM homeostasis and tumour invasion as demonstrated in breast and prostate cancer cells [30], [31] and oesophageal adenocarcinoma cells made to over-express uPA *in vitro* [11]. Elevated levels of uPA and PAI can also enhance migration, through modulation of cell-to-matrix adhesion [7], [32]. The involvement of PAI and uPA in invasion may explain the functional significance of the inhibition of kinase pathways on the invasive properties of oesophageal adenocarcinoma cells (Figure 8). It is worth noting that OE33 was the only cell line that had a demonstrable increase in the SMAD reporter construct as well as increased kinase activity in response to TGFβl. For this cell line it is not possible to rule out a contribution of Smad activation in the TGFβl response of OE33 cells although the invasive properties were significantly reduced by kinase inhibitors (Figure 8).

uPA and PAI expression through the activity of ERK and JNK may also be relevant for tumour progression *in vivo*. For example, in prostate, breast and pancreatic carcinoma cells, JNK, or the upstream activator of both kinases, MKK4, has been demonstrated to mediate growth of cells *in vitro*, and as xenografts *in vivo* [33], [34]. Furthermore, over-expression of PAI and uPA has been shown to correlate strongly with tumour stage and to be of independent prognostic significance in oesophageal adenocarcinoma [13], [35].

In conclusion, we have shown that there is a divergent response to TGFβ in cells from BE adenocarcinoma. The Smad dependent anti-proliferative response is lost; however, the ability of TGFβ to induce ECM genes PAI and uPA prevails. This occurs predominantly through the ERK and JNK MAPK pathways, and thus contributes to the invasive properties of the oesophageal tumour cell. Such a differential response utilising different cell signalling pathways may allow TGFβ to play a role in both the initiation and progression of oesophageal adenocarcinoma.

## Materials and Methods

### Cell lines and culture conditions

For this study cell lines derived from Barrett's adenocarcinoma were used. Barrett's associated adenocarcinoma cell lines OE33 (European Collection of Cell Cultures, Wiltshire, UK), TE7 (gift from T Nishihira, Kurokawa County Hospital, Japan), were maintained in RPMI-1640, and SEG, BIC and FLO (gift from D Beer, University of Michigan, MI, USA) were maintained in DMEM. All cells were supplemented with 10% (FBS), 100 U/mL penicillin, 100 mg/mL streptomycin and 2 mM glutamine.

### Kinase inhibition

Cells were grown to 70% confluence in complete medium, which was then replaced with serum free medium for 3 h. Cells were pre-treated for 1 h with inhibitors at the minimal concentration leading to kinase inhibition (data for determining optimal inhibitor concentrations not shown): PI3K inhibitors - Wortmannin (5 nM) and LY294002 (20 µM) (Calbiochem-Novabiochem, Nottingham, UK); ERK inhibitors - U0126 (inhibits MEK 1 and 2), (10 µM) and PD098059 (inhibits MEK 1), (20 µM) (Sigma-Aldrich, Dorset, UK); and JNK inhibitors - SP600125 (20 µM) (Sigma-Aldrich) and curcumin (10 µM) (Calbiochem-Novabiochem). Cells were then treated with 10 ng/mL TGFβ1 for 24 h in the presence of inhibitors. This concentration was used as we have previously shown in dose response experiments that it is the optimum concentration to induce a decrease in proliferation [17].

### Flow cytometry

10^4^ cells were seeded in 6 well plates in complete growth medium, and all cells were synchronised overnight in serum-free medium. Cells were then either incubated in complete medium with or without 10ng/mL recombinant TGFβ1 (Tebu-bio, Cambridgeshire, UK), or kept in serum free medium, for 24 h. Cells were harvested, fixed in 70% ethanol at 4°C and then incubated in PBS, 0.1% Tween 20 containing 2 µg/mL propidium iodide (Sigma-Aldrich) and 200 µg/mL RNAse A for 30 min at 37°C. DNA content was analysed by fluorescence associated cell sorting (FACS) using the BD LSR1 flow cytometer (BD Biosciences, Oxford, UK), and data captured using Cell quest software (BD Biosciences, Oxford, UK).

### Immunofluorescence staining

Cells were seeded overnight onto sterilised coverslips in a 24 well plate. Media was removed and replaced with fresh media with or without 10 ng/ml TGFβ1. Following a 6 h incubation, media was removed and the coverslips washed twice in 0.5 ml PBS. The coverslips were then stained with Smad2/3 antibody (clone E-20, Santa Cruz Biotechnology, Inc., Santa Cruz, CA, USA) following a procedure described previously [17].

### Luciferase reporter assay

Cells were seeded in 24 well plates at a density of 5×10^4^ cells/well and left overnight. Using Fugene6 transfection reagent (Roche Applied Science, East Sussex, UK) cells were transfected with the reporter plasmid (CAGA)_12_-Luc, containing 12 repeats of the CAGA Smad3/4 binding sequence. Cells were also transfected with Renilla luciferase reporter plasmid to normalise transfection efficiency. After 24 h medium was replaced with serum free medium with or without TGFβ1 (10 ng/mL). After a further 24 h luciferase activity was determined using the Dual Luciferase Reporter System (Promega, Southampton, UK) in a TD 20/20 Luminometer (Promega).

### Quantitative real time PCR

Two micrograms of total RNA isolated using Trizol Reagent (Invitrogen, Paisley, UK) were reversed transcribed, and 1 µL of cDNA was amplified in a 50 µl volume containing 25 µL of 2× Quantitect SYBR Green PCR Master Mix (Qiagen, West Sussex, UK) and 0.2 µM of each primer (Table 3). Triplicate reactions were performed in a DNA Opticon using the conditions of initial enzyme activation of 15 mins at 95°C, followed by 30 to 35 cycles of 10 s at 95°C, 20 s at the annealing temperature and 20 s at 72°C. The melting curve was constructed for each primer to ensure reaction specificity. Following PCR, the threshold cycle (C_T_) was obtained and relative quantities determined compared to β actin.

**Table 3:**
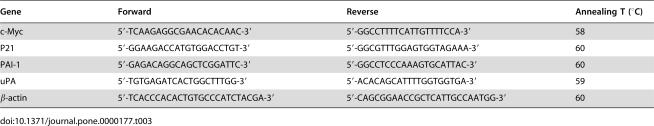
Primer sequences

Gene	Forward	Reverse	Annealing T (°C)
c-Myc	5′-TCAAGAGGCGAACACACAAC-3′	5′-GGCCTTTTCATTGTTTTCCA-3′	58
P21	5′-GGAAGACCATGTGGACCTGT-3′	5′-GGCGTTTGGAGTGGTAGAAA-3′	60
PAI-1	5′-GAGACAGGCAGCTCGGATTC-3′	5′-GGCCTCCCAAAGTGCATTAC-3′	60
uPA	5′-TGTGAGATCACTGGCTTTGG-3′	5′-ACACAGCATTTTGGTGGTGA-3′	59
β-actin	5′-TCACCCACACTGTGCCCATCTACGA-3′	5′-CAGCGGAACCGCTCATTGCCAATGG-3′	60

### Western blotting and immunoprecipitation

Immunoprecipitation was required due to the low sensitivity of western blotting alone for the phosphorylated forms of AKT, ERK and JNK. Protein lysates were prepared with ice-cold lysis buffer (20 mM Tris pH 7.8, 150mM NaCl, 1 mM EDTA, 1% NP40) containing protease inhibitors (Complete tablet, Roche, Germany). 200 µg of protein was immunoprecipitated with 1 µg ERK1/2 antibody (Santa Cruz Biotechnology, Inc., Santa Cruz, CA, USA), JNK antibody (1∶100) (New England Biolabs, Herts, UK) and Akt antibody (1∶1000) (New England Biolabs, Herts, UK). 20*μ*L of immunoprecipitated sample or 50 µg of proteins were then separated by gel electrophoresis on 8% polyacrylamide gels and transferred to a PVDF membrane (Hybond-P, Amersham Biosciences, Amersham, UK). Membranes were incubated overnight at 4°C with the following antibodies: phospho-ERK1/2 (0.1 µg/mL) (Calbiochem-Novabichem), phospho-JNK (1∶1000) (Calbiochem-Novabichem), phospho-Akt (New England Biolabs) or pSmad2/3 (Santa Cruz Biotechnology Inc., Santa Cruz, CA, USA). Membranes were incubated with a 1∶10,000 dilution of peroxidase conjugate secondary IgG antibody (Perbio Science, Cheshire, UK), and signals detected by chemiluminescence (ECL, Amersham Biosciences, Amersham, UK).

### Zymography and reverse zymography

To assess uPA activity 50 µg of protein lysate were incubated with non-reducing buffer for 20 min at 37°C, then electrophoresed in a polyacrylamide gel containing 2 mg/mL α-casein (Sigma-Aldrich) and 5 µg/mL plasminogen (Sigma-Aldrich). Gels were washed 2 times for 30 min each in 50 mM Tris-HCl (pH 7.6), 2.5% Triton X-100, incubated for 16 h at 37°C in 50 mM Tris-HCl (pH 7.6) and then stained for 1 h in a solution of 30% methanol, 10% acetic acid and 0.5% Coomassie brilliant blue G250 and de-stained. Urokinase activity was detected as digested clear bands on a blue background. To determine PAI activity lysates were subjected to SDS-PAGE in gels containing 2 mg/mL α-casein, 5 µg/mL plasminogen and 0.8 U/mL human urokinase (Calbiochem-Novabichem). Gels were then washed and stained as above. PAI activity was detected as dark undigested bands against a light background.

### Wound healing assay

Cells were grown in a 60 mm dish to confluency, and then wounded using a hand held engraving drill (Minicraft MB168) fitted with a cut down 10 µL pipette tip. Wound area was measured using an eyepiece graticule and calibrated using a haemocytometer grid, at 10× magnification. The wounded monolayer was washed with PBS, and then cultured in serum free medium, containing 10 ng/mL TGFβ1 alone or with LY294002 (20 µM), PD98590 (20 µM) or SP600125 (20 µM) for 24 h. The wound area was again measured, the absolute change in wound size deduced and the % healing at 24 h then calculated. In each experiment 8 wounds were made and each experiment was repeated at least three times.

### Invasion assay

Fifty µL of a 10 mg/mL solution of matrigel (BD Biosciences), containing 25 µg/mL plasminogen was applied to the upper compartment of a Transwell 24 well insert (BD Biosciences) of 8 µm pore size, and incubated at 37°C. Cells were washed three times in serum free RPMI, and then 1×10^5^ cells were resuspended in 200 µl of serum free RPMI containing 10 ng/mL TGFβ1 alone or with LY294002 (20 µM), PD98590 (20 µM) or SP600125. Cells were then applied to the upper compartment of the invasion chamber. In the lower compartment 500 µl of serum containing medium was added and the cells were then incubated at 37°C for 24 h. Medium was then removed and the cells were then fixed for 20 min in methanol at −20°C. Chambers were stained in haematoxylin for 1 min and a cotton swab used to remove non-invasive cells in the upper compartment. Membranes were excised from the chamber and fixed on a microscope slide. Membranes were quantified by counting the number of cells that had invaded the lower chamber in 5 high-power fields.[11]

### Slow aggregation assay

100 µL of agar was solidified in 96 well plate formats. Cells were washed with Moscona's solution, trypsinised and then resuspended in serum free media. 2×10^4^ cells (100 µl) were seeded into each well and left for 1 h. 100 µL of serum free medium containing TGFβ1 alone or with LY294002 (20 µM), PD98590 (20 µM) or SP600125 (20 µM). [36] Aggregates, for 10 replicates in three independent experiments, were scored after 24 h incubation at 37°C, 5% CO_2 _using an inverted microscope. Replicates were scored 0 if no aggregates were present, 1 for small aggregates and 2 for large aggregates.

### Statistics

The Kruskal-Wallis test was used to compare values between different data sets, and the Dunn's Multiple Comparison Test was used to identify specific differences, using Graphpad Prism software. A p<0.05 was necessary for statistical significance.

## Supporting Information

Table S1 (0.04 MB DOC)

Figure S1 Densitometry analysis of phosphorylated MAPK. The western blots (as presented in figure 5) were analysed by densitometry. The data is presented as fold increase in density of the TGFb treated band compared to the untreated control for each cell line and each MAPK.
(10.47 MB TIF)

Figure S2 Densitometry analysis of uPA and PAI activity. The zymographies (as presented in figure 7) were analysed by densitometry. The data is presented as fold increase in density of the TGFb treated band with inhibitors compared to the TGFb treated control for each cell line and each MAPK.
(9.82 MB TIF)
