# Supplementary material for: Selective Loss of TGFβ Smad-Dependent Signalling Prevents Cell Cycle Arrest and Promotes Invasion in Oesophageal Adenocarcinoma Cell Lines
Source: PLoS One. 2007 Jan 31;2(1):e177. doi: 10.1371/journal.pone.0000177 (PMC1766472; doi:10.1371/journal.pone.0000177)
Supplement: Table S1 — (0.04 MB DOC) [file pone.0000177.s001.doc]

# Supplementary table 1

TGFβ induced modulation of PAI and uPA expression

|  | **OE33** | **TE7** | **SEG** | **BIC** | **FLO** |
| --- | --- | --- | --- | --- | --- |
| **PI3K inhibition** |  |  |  |  |  |
| PAI-1 mRNA (fig 6A) | Wo, LY | - | Wo, LY | NP | - |
| PAI-1 enzyme activity (fig 7) | Yes | Yes | Yes | NP | Yes |
| uPA mRNA (fig 6B) | - | - | Wo, LY | NP | Wo, LY |
| UPA enzyme activity (fig 7) | Some | Some | Yes | NP | Yes |
|  |  |  |  |  |  |
| ERK inhibition |  |  |  |  |  |
| PAI-1 mRNA (fig 6A) | PD, U | PD | PD, U | NP | PD |
| PAI-1 enzyme activity (fig 7) | Yes | Yes | Yes | NP | Yes |
| uPA mRNA (fig 6B) | PD | PD | PD | NP | PD |
| UPA enzyme activity (fig 7) | Some | Some | Yes | NP | Yes |
|  |  |  |  |  |  |
| JNK inhibition |  |  |  |  |  |
| PAI-1 mRNA (fig 6A) | SP, Cu | SP, Cu | SD, Cu | NP | SP, Cu |
| PAI-1 enzyme activity (fig 7) | Yes | Yes | Yes | NP | Yes |
| uPA mRNA (fig 6B) | - | SP, Cu | - | NP | SP, Cu |
| UPA enzyme activity (fig 7) | Yes | Yes | Yes | NP | Yes |

Yes/No/Some - qualitative assessment

– no significant quantitative change

W (wortmaninn), LY (LY294002), PD (PD098059), U (U0126), SP (SP600125) and Cu (Curcumin) indicate that significance was reached for the named inhibitor.

NP experiments not performed
