# Supplementary figures and images for: Selective Loss of TGFβ Smad-Dependent Signalling Prevents Cell Cycle Arrest and Promotes Invasion in Oesophageal Adenocarcinoma Cell Lines
Source: PLoS One. 2007 Jan 31;2(1):e177. doi: 10.1371/journal.pone.0000177 (PMC1766472; doi:10.1371/journal.pone.0000177)

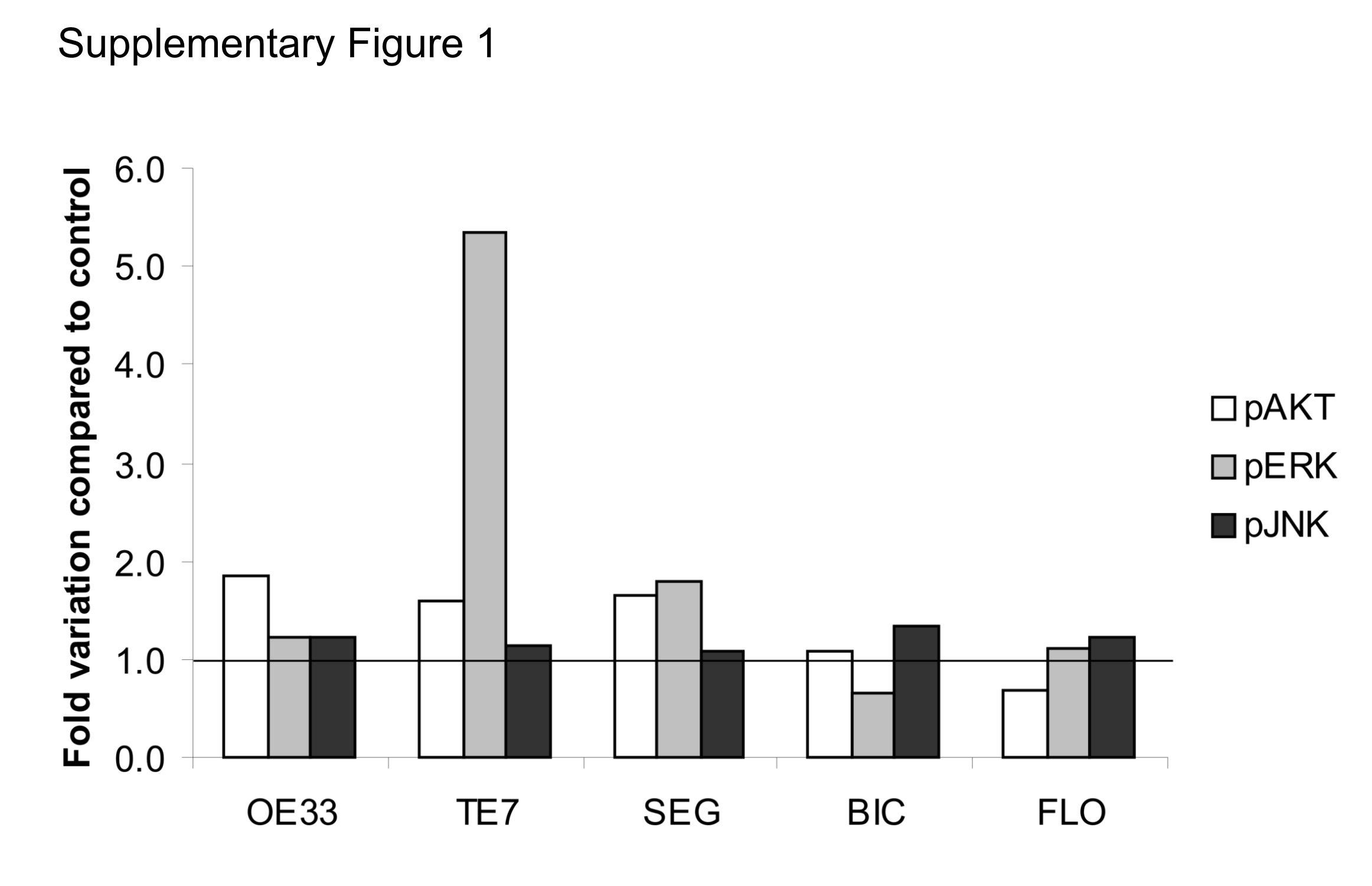

Supplement: Figure S1 — Densitometry analysis of phosphorylated MAPK. The western blots (as presented in figure 5) were analysed by densitometry. The data is presented as fold increase in density of the TGFb treated band compared to the untreated control for each cell line and each MAPK. (10.47 MB TIF) [file pone.0000177.s002.tif]

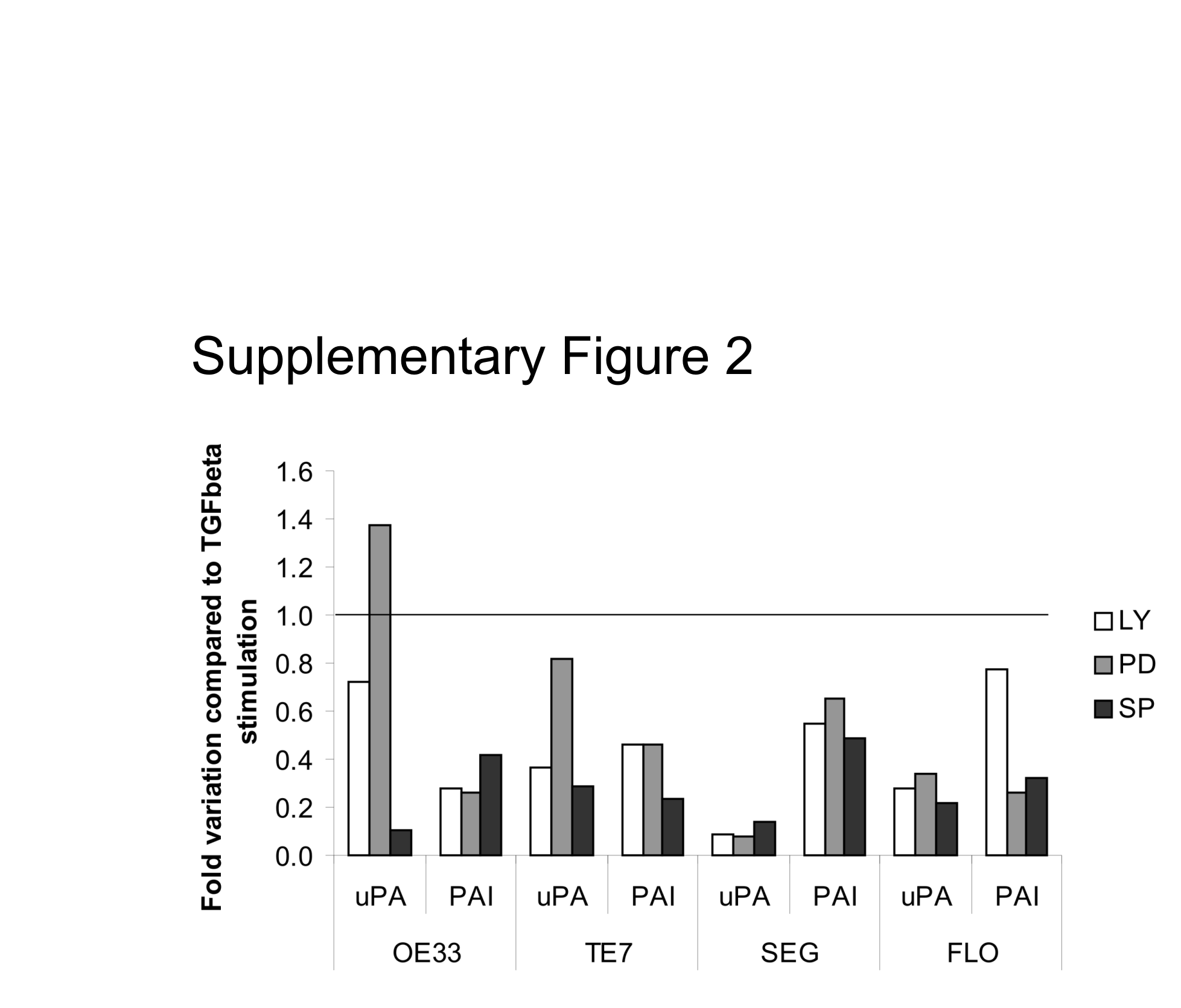

Supplement: Figure S2 — Densitometry analysis of uPA and PAI activity. The zymographies (as presented in figure 7) were analysed by densitometry. The data is presented as fold increase in density of the TGFb treated band with inhibitors compared to the TGFb treated control for each cell line and each MAPK. (9.82 MB TIF) [file pone.0000177.s003.tif]
